# Grisel's Syndrome: A Rare Complication following Adenotonsillectomy

**DOI:** 10.1155/2014/703021

**Published:** 2014-03-24

**Authors:** Abdulkadir Bucak, Sahin Ulu, Abdullah Aycicek, Emre Kacar, Murat Cem Miman

**Affiliations:** ^1^Department of Otolaryngology, Faculty of Medicine, Afyon Kocatepe University, Ali Cetinkaya Kampusu, Tıp Fakultesi Izmir Karayolu, 03200 Afyonkarahisar, Turkey; ^2^Department of Radiology, Faculty of Medicine, Afyon Kocatepe University, Ali Cetinkaya Kampusu, Tıp Fakultesi Izmir Karayolu, 03200 Afyonkarahisar, Turkey; ^3^Department of Otolaryngology, Faculty of Medicine, Izmir University, Yeni Girne Bulvarı, 1825 Sok, No. 12, Karsıyaka, 35350 Izmir, Turkey

## Abstract

Grisel's syndrome is a nontraumatic atlantoaxial subluxation which is usually secondary of an infection or an inflammation at the head and neck region. It can be observed after surgery of head and neck region. Etiopathogenesis has not been clearly described yet, but increased looseness of paraspinal ligament is thought to be responsible. Patients typically present with painful torticollis. Diagnosis of Grisel's syndrome is largely based on suspicion of the patient who has recently underwent surgery or history of infection in head and neck region. Physical examination and imaging techniques assist in diagnosis. Therefore, clinicians should be aware of acute nontraumatic torticollis after recently applied the head and neck surgery or undergone upper respiratory tract infection. In this paper, a case of an eight-year-old male patient who had Grisel's syndrome after adenotonsillectomy is discussed with review of the literature.

## 1. Introduction

Grisel's syndrome that is known as the subluxation of atlas and axis was firstly described by Sir Charles Bell in 1830 in a syphilitic patient with pharyngeal ulceration. Bell reported death in this patient by spinal cord compression due to atlanto-axial subluxation and autopsy report showed the erosion of the axis transverse ligament [1–3]. In 1930, the French physician Grisel who gave his name to the syndrome reported 2 cases having this syndrome which had developed after nasopharyngeal inflammation [2–4]. Increased flexibility of the atlantoaxial joint ligaments has been implicated as the underlying reason for this syndrome [1–3, 5]. Although the etiopathogenesis of this syndrome has not been exactly proven, pediatric age group, history of pharyngitis, adenotonsillitis, abscess of peritonsillar and cervical region, otitis media, trauma, any upper respiratory tract infection, genetic disorders, and to-be-performed head and neck surgery were reported as risk factors. There is not a definite consensus criterion for the diagnosis and treatment, but early diagnosis is very important for prognosis. Late diagnosis and inadequate treatment may cause neurological sequelae and/or painful and lasting deformity of the neck [2, 5]. Acute form can be easily treated with bed rest, antibiotics, anti-inflammatory agents, immobilization, and/or simple traction; some acute cases and almost all of the chronic cases (last longer than a month) cannot be conservatively treated. The treatment of these patients may also require surgical intervention that may include skeletal traction or bone fusion [5]. In this paper, a case of an eight-year-old male patient who had Grisel's syndrome after adenotonsillectomy is discussed with review of the literature.

## 2. Patient

**Figure 1:**
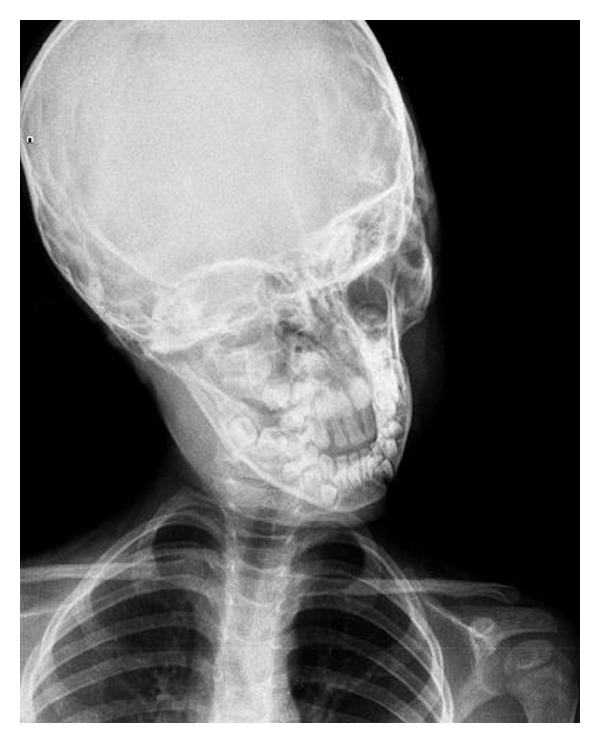
Cock-robin position on anterior-posterior direct cervicography.

**Figure 2:**
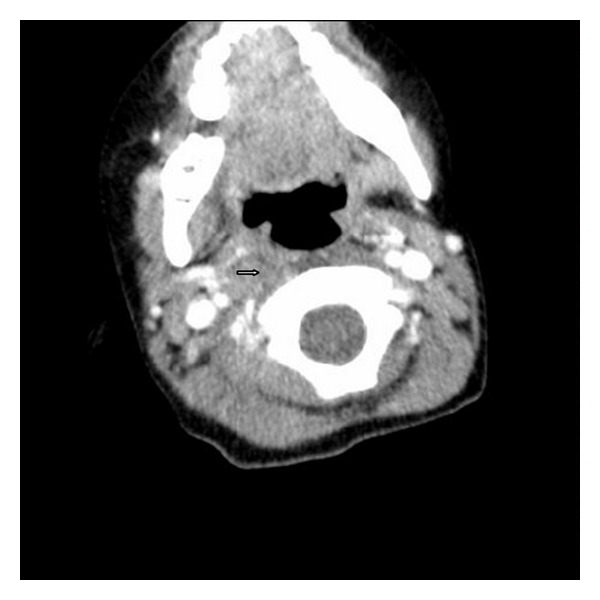
Asymmetric thickening of the right parapharyngeal soft tissue (arrowhead).

**Figure 3:**
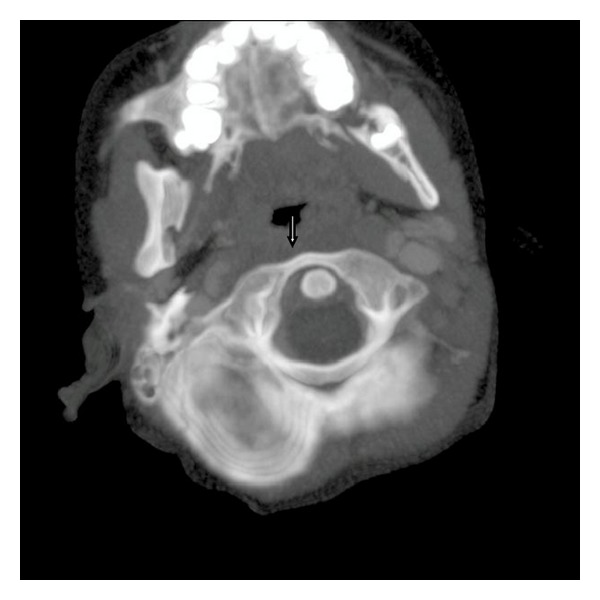
Rotator atlantoaxial subluxation to soft tissue asymmetry (arrowhead).

An 8-year-old male patient who had adenotonsillectomy was admitted with painful torticollis on the postoperative fourth day. Any complication related to the surgery has not been detected on the examination. Neurological examination was normal. Direct cervicography (DCG) and cervical computerized tomography (CT) were performed for diagnosis. Cock-robin position was detected on anterior-posterior DCG (Figure 1). Asymmetric thickening of the right parapharyngeal soft tissue and rotator atlantoaxial subluxation through the asymmetric thickening of soft tissue were observed on cervical CT (Figures 2 and 3). The patient was assessed by physical therapy and rehabilitation and neurosurgery department and recommended absolute bed rest and immobilization with a cervical collar. Antibiotic (amoxicillin-clavulanic acid) and anti-inflammatory (ibuprofen) treatment were given to the patient. The symptoms gradually decreased by treatment and it took 4 weeks of full recovery. Antibiotic therapy was continued to the 14th day and the anti-inflammatory therapy was continued to 28th day.

## 3. Discussion

Grisel's syndrome is atlantoaxial joint subluxation without trauma or concomitant bone pathology, generally occurs in childhood and etiopathogenesis has not been clearly explained. According to the recent literature, vascular plexus providing the drainage of posterosuperior pharyngeal area is responsible. Periodontoid plexus is connected with posterior nasopharyngeal veins via pharyngovertebral vein. Any infective embolism may spread from superior pharyngeal area to upper cervical joints due to this plexus which has not any lymph node [5, 6]. Primary stabilizer is transverse; secondary stabilizer is alar ligament for atlantoaxial joint [5]. Inflammatory mediators cause synovial and vascular congestion, periligamentous inflammation, and oedema, resulting in subluxation and finally, may progress to imbalance of cervical spine with complicated neurological outcomes [2, 5].

Fielding has described 4 types of atlantoaxial subluxations [7]. The first type is the rotation of atlas above axis without anterior displacement. The second type is the rotation of atlas above lateral reticular process with 3–5 mm anterior displacement. The third type is the rotation of atlas with anterior dislocation more than 5 mm and the fourth type is the rotation of atlas with posterior dislocation [4, 7, 8]. Types 1 and 2 of subluxation are most common and neurological symptoms are not available. Our patient had type 1 atlantoaxial subluxation and neurological symptoms have not been detected. The spinal cord compression and severe neurological findings can be seen in types 3 and 4. In the literature, the patients having Grisel's syndrome were presented with atlantooccipital, c (cervical) 2-3 and c 3-4 subluxation [1, 4–6, 9, 10]. Although Grisel's syndrome is seen in children with a very large extent, it can be seen in adult patients too. Looseness of the ligament between c1 and c2 vertebrae (especially cross ligaments) is more apparent in children [3]. Grisel's syndrome is generally reported in patients between ages 5 and 12 years and gender dominance has not been detected [1, 2]. Of the patients with Grisel's syndrome, 90% of them are smaller than 21 years of age and 68% are under 12 years [3]. Our patient's age (8 years old) was compatible with the literature.

Patients classically present with neck stiffness and torticollis-associated painful neck movements. The main complaint of our patient was torticollis. This syndrome can be seen after rhinopharyngitis, cervical osteomyelitis, rheumatic conditions, and surgical procedures such as adenoidectomy, tonsillectomy, repairment of choanal atresia, and mastoidectomy. Also, hard object shock, mumps, or retropharyngeal abscess have been reported [1, 2, 4, 5]. It can be seen with congenital syndromes such as Down and Marfan which have increased ligament looseness [1, 3]. Another factor of interest is increased atlantoaxial distance in Down's syndrome [4]. The other risk patient groups are characterized by spinal instability such as Morquio's syndrome, Klippel-Feil syndrome, osteogenesis imperfecta, and neurofibromatosis. Other pathologies with similar symptoms must be ruled out as like the cervical bone anomalies, posterior fossa tumors, or cervical trauma [3, 11].

The 67% of patients with Grisel's syndrome were seen after surgery with reference to some authors, contrary to this at the another paper including 103 patients for 55 years; 48% were secondary to infection and 40% were secondary to surgery. The 78% of patients who were secondary to surgery were postadenotonsillectomy [4, 12]. In our opinion, the provoking factors were surgery (adenotonsillectomy) and inflammation in our case.

Torticollis can spontaneously occur after pharyngitis and minor trauma to the neck; the differential diagnosis of torticollis requires a complete medical history, clinical and radiological examination. History of disease is of important for medical history, predisposing factors, and recurrence [5]. It should be kept in mind that the neck pain, limited neck motion, cock-robin position, and torticollis can be early signs of Grisel's syndrome after adenoidectomy and/or tonsillectomy. “The expected situation after surgery” should be considered like general opinion.

Sensitive palpable c2 spinous process is a strong indicator of atlantoaxial subluxation (Sudeck's sign) [4, 11, 13]. Patients may feel neck pain and tingling at the upper and lower extremities in the neck flexion (Lhermitte's sign) [13]. Although less than 15% of patients show neurological signs and symptoms, extreme consequences such as quadriplegia and sudden death might be seen [2]. Radiological evaluation is very important for early diagnosis because increased atlantoodontoid distance could be detected in the lateral projection. Normal atlantoodontoid distance is ≤3 mm in adults and ≤5 mm in children [8]. CT and magnetic resonance imaging (MRI) are excellent diagnostic tools to show the deep neck infection and relationship between ligamentous and bone structure of the spine [4]. The 3D (dimensional) CT could provide a detailed assessment of the cervical spine and subluxation [11, 13]. Dynamic CT can cause neurological complications [5]. Inflammatory indicators are not specific for this syndrome [4].

If effective and immediate treatment does not commence, subluxation can develop [3]. Consultation of the patient with relevant branches (physical medicine and rehabilitation, neurosurgery and pediatrics, etc.) and evaluation is very important for without-delay treatment and preventing significant neurological deficits [4]. Treatment must be quickly initiated to avoid neurological complications [5]. Conservative treatment including bed rest, antimicrobial therapy, muscle relaxants, anti-inflammatory agents, external fixation, cervical traction, and soft or hard collar is of great importance at this early period which has rotational deformity without subluxation [4, 8]. The subluxation can develop after painful torticollis and/or fever in patients. If muscle spasm lasts longer than 24 hours, diazepam can be added to treatment and early treatment can prevent the poor prognosis.

In conclusion, diagnosis of the Grisel's syndrome is largely based on suspicion of the patient who has recently underwent surgery or history of infection in head and neck region [4, 5]. Early diagnosis of the atlantoaxial subluxation is required for careful clinical and radiological evaluation and consultation with relevant branches. Early intervention is critical for prognosis; conversely, delay in diagnosis can be dramatic. Therefore, clinicians should be aware of acute nontraumatic torticollis after recently applying the head and neck surgery [5].
